# Neutral Sphingomyelinase in Physiological and Measles Virus Induced T Cell Suppression

**DOI:** 10.1371/journal.ppat.1004574

**Published:** 2014-12-18

**Authors:** Nora Mueller, Elita Avota, Lena Collenburg, Heike Grassmé, Sibylle Schneider-Schaulies

**Affiliations:** 1 University of Würzburg, Institute for Virology and Immunobiology, Wuerzburg, Germany; 2 Institute for Molecular Biology, Essen, Germany; St. Jude Children's Research Hospital, United States of America

## Abstract

T cell paralysis is a main feature of measles virus (MV) induced immunosuppression. MV contact mediated activation of sphingomyelinases was found to contribute to MV interference with T cell actin reorganization. The role of these enzymes in MV-induced inhibition of T cell activation remained equally undefined as their general role in regulating immune synapse (IS) activity which relies on spatiotemporal membrane patterning. Our study for the first time reveals that transient activation of the neutral sphingomyelinase 2 (NSM2) occurs in physiological co-stimulation of primary T cells where ceramide accumulation is confined to the lamellum (where also NSM2 can be detected) and excluded from IS areas of high actin turnover. Genetic ablation of the enzyme is associated with T cell hyper-responsiveness as revealed by actin dynamics, tyrosine phosphorylation, Ca^2+^-mobilization and expansion indicating that NSM2 acts to suppress overshooting T cell responses. In line with its suppressive activity, exaggerated, prolonged NSM2 activation as occurring in co-stimulated T cells following MV exposure was associated with aberrant compartmentalization of ceramides, loss of spreading responses, interference with accumulation of tyrosine phosphorylated protein species and expansion. Altogether, this study for the first time reveals a role of NSM2 in physiological T cell stimulation which is dampening and can be abused by a virus, which promotes enhanced and prolonged NSM2 activation to cause pathological T cell suppression.

## Author Summary

Though the ability of measles virus (MV) to impair T cell activation has long been known, it is mechanistically not well understood. We have shown earlier that MV can contact dependently trigger activation of sphingomyelinases which is known to affect compartmentalization of membrane lipids and proteins. Because these are particularly important in the activity of the immune synapse (IS), we investigated whether MV-induced sphingomyelinase activity would interfere at that level with T cell activation. Our study for the first time revealed that the neutral sphingomyelinase 2 (NSM2) is transiently activated in primary T cells by co-stimulation through CD3 and CD28, and that this does occur to dampen early T cell responses. The virus appears to exploit this inhibitory activity of the enzyme to suppress T cell activation by promoting an enhanced and prolonged NSM2 activation. These findings do not only assign a hitherto novel role of the NSM2 in regulating T cell responses, but also reveal a novel strategy for viral T cell suppression.

## Introduction

Plasma membrane ceramides are released in response to activation of sphingomyelinases and condense into large platforms which alter biophysical properties of the cell membrane. In addition to other stimuli, ligation of certain surface molecules, also including death receptor family members and viral attachment receptors, efficiently activates neutral and/or acid sphingomyelinase (NSM or ASM, respectively) followed by ceramide release (reviewed in [1]–[3]). Ceramide enriched membrane microdomains act to regulate sorting of membrane proteins and their signalosomes, and this affects a variety of biological responses including lateral and vertical receptor segregation as particularly relevant for pathogen uptake, apoptosis, cell motility and proliferation [3]–[6].

Measles virus (MV) causes profound generalized immunosuppression and interference with T cell viability, expansion and function is one of its major hallmarks. A plethora of findings supports the interpretation that MV is acquired and transferred by CD150+ antigen-presenting cells to the secondary lymphatic tissues where it can be transmitted to and deplete CD150+ lymphocytes, especially memory T cells [7]–[9]. Though being infected to a very limited extent, peripheral blood cells of patients, however, are generally refractory to expansion driven by polyclonal and antigen-specific stimulation, implying they had been paralysed by mechanisms independently of direct infection. In line with this hypothesis, exposure of uninfected lymphocytes to UV-inactivated MV or the MV glycoprotein complex (gpc) was sufficient to induce their arrest *in vitro* and *in vivo* [10]–[13]. For this, the gpc interacts with an as yet unknown receptor on the surface of T cells (which is not identical to CD150 [14]) to abrogate relay of T cell receptor (TCR) signaling at the level of the phosphatidyl-inositol-phosphate-3-kinase (PI3K) and its downstream effectors, Akt kinase, Vav1, Rac1 and Cdc42 [15]–[17]. Because these are also major regulators of actin cytoskeletal dynamics, MV contact induced physical T cell paralysis is reflected by collapse of actin based protrusions and loss of polarity and motility on fibronectin [15]–. Thus, by targeting the PI3K, MV abrogates activation of downstream effectors essentially mediating S-phase progression, but also actin dynamics which is of key importance in organizing the functional architecture of the immune synapse (IS) where T cell signaling is initiated and sustained [19].

In order to interfere with TCR signaling, MV has to initiate signaling upon binding to T cells itself. This involved sequential activation of NSM2 (the NSM species abundant at the plasma membrane) and ASM, which almost entirely accounted for MV interference with actin cytoskeletal integrity and dynamics [18], though the role of sphingomyelinase activation on T cell activation remained unknown.

The role of ceramide release in regulating T cell activation by CD3/CD28 co-stimulation is unclear. Ligation of either CD3 or CD28 alone caused activation of NSM or ASM, respectively [20], [21]. CD3-mediated NSM activation was dispensable for TCR-induced overall tyrosine phosphorylation, however, required for IL-2 production and MAPK activation [20], while ASM-mediated ceramide release was important for CD28-dependent NF-κB activation [21]. In the latter study, exogenous supply of ceramides fully replaced ASM activity in co-stimulation. In contrast, other studies indicated an inhibitory activity of ceramide in co-stimulating CD3 signaling [22]–[24], and ceramide metabolites are of low abundance in CD3 associated domains immuno-isolated from T cells following activation [25]. This indicates that sphingomyelin breakdown, if occurring at all, has to be tightly controlled and compartmentalized at the level of the IS, and, if aberrantly induced, might translate into T cell inhibition.

With the present study we addressed activation of sphingomyelinases and subcellular distribution of ceramide accumulation upon CD3/CD28 co-ligation. Strikingly, co-stimulation entirely abrogated ASM activation while caused an early rise in NSM activity. This proved to be important for dampening thresholds of T cell activation because cell spreading as required for formation of IS interaction platforms and overall tyrosine phosphorylation were initiated earlier and were enhanced upon NSM knockdown as was expansion of T cells. Enhancement of sphingomyelinase activity upon pre-exposure to MV was associated with accumulation of ceramide enriched membrane domains at the synaptic interface. Importantly, MV induced NSM activation substantially accounted for loss of spreading responses and partially for MV interference with TCR-induced tyrosine-phosphorylation and expansion. Altogether, our findings indicate that the ability of NSM to dampen the threshold of T cell activation is exploited by MV for T cell suppression.

## Results

### Sphingomyelinase activation and ceramide release are tightly controlled in T cell co-stimulation

**Figure 1:**
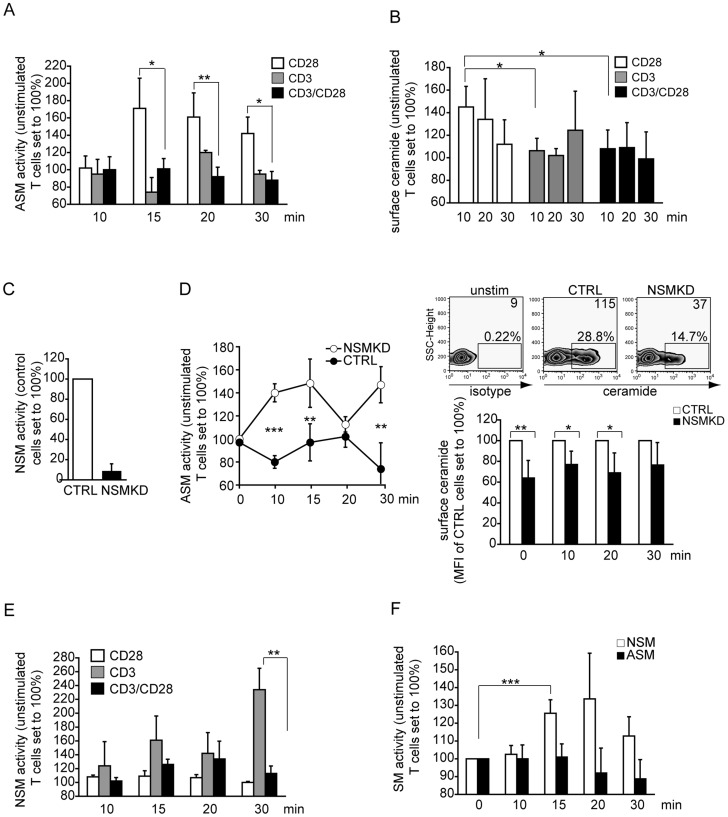
ASM activation is ablated, while NSM activity is induced early in co-stimulated primary human T cells. A. ASM activity was determined in membrane extracts of T cells activated by α-CD28, α-CD3 or α-CD3/CD28 over time. B. Surface ceramide was detected on T cells activated by ligation of CD3, CD28 or CD3/CD28 by flow cytometry C. NSM activity in membrane extracts of T cells 96 h following transfection of control (CTRL) or NSM2 siRNA (NSMKD). D. ASM activity in membrane extracts of αãCD3/CD28 co-stimulated T cells (left) and extrafacial ceramide display (FACS staining) (right, upper graphs: unstimulated, CTRL or NSMKD T cells, upper right corner values represent mean fluorescence intensities; bottom graph: kinetics of ceramide surface display on CTRL versus NSMKD co-stimulated T cells. E. NSM activity was determined in membrane extracts of T cells activated by α-CD28, α-CD3 or α-CD3/CD28. F. ASM and NSM activity were determined over time in α-CD3/CD28 co-stimulated T cells.

CD28 ligation causes ASM activation followed by ceramide release [21], which can be inhibitory to T cell stimulation [23], [24]. We therefore investigated whether CD3/CD28 co-stimulation would also promote ASM activation in primary T cells. Using a planar system (plate bound antibodies) for stimulation, ligation of CD28 alone (but not that of CD3) expectedly induced ASM activity which peaked after 15 min. This, was, however, completely abrogated upon CD3/CD28 co-stimulation where ASM activity did not significantly increase during the observation period (Fig. 1A). Activated ASM is the major source of extrafacial ceramide, and consequently, ceramide was displayed at the cell surface following ligation of CD28, but not of CD3 or CD3/CD28 co-ligation on T cells as detected by flow cytometry. This again supported the interpretation that CD28-mediated ASM activation is abolished upon co-stimulation (Fig. 1B, and S1A Fig.). Because NSM2 activation after CD3 ligation and cross-regulation of sphingomyelinases have been described earlier [18], [20], [26], we analyzed whether NSM2 would be involved in ASM inhibition by knocking down NSM2 in T cells by specific siRNA (further referred to as NSMKD T cells) (Fig. 1C). In contrast to what has been reported for other cell types [26], NSM knockdown did not augment basal ASM activity (S1B Fig.). As measured in co-stimulated untransfected T cells (Fig. 1A), the ASM activity remained at or below basal levels in control siRNA transfected cells (further refered to as CTRL T cells) (Fig. 1D, left panel). In NSMKD T cells, biphasic activation of the enzyme occurred to levels comparable to those measured upon CD28 ligation (Fig. 1A and 1D, left panel) indicating that NSM activity was required to extinguish that of ASM in co-stimulation. ASM activity was enhanced in NSMKD T cells, however, accumulation of extrafacial ceramides was not and rather remained at levels below that of CTRL T cells (Fig. 1D, right panel). In line with previous findings [20], early NSM activation in response to CD3 ligation did occur in untransfected T cells, and did not significantly differ from that induced upon co-stimulation, while a late peak of NSM activation was only seen after CD3 ligation (Fig. 1E). As revealed by direct comparison of activities of both enzymes, ASM activation does not occur in co-stimulated T cells while NSM was transiently activated clearly exceeding background levels, retained within 15 minutes and dropped thereafter (Fig. 1F). To the best of our knowledge, cross-regulation of ASM and NSM upon co-stimulation of receptors individually promoting their activation has not been previously observed.

### Ceramide enriched membrane domains are confined to the lamellum where NSM2 is co-detected

**Figure 2:**
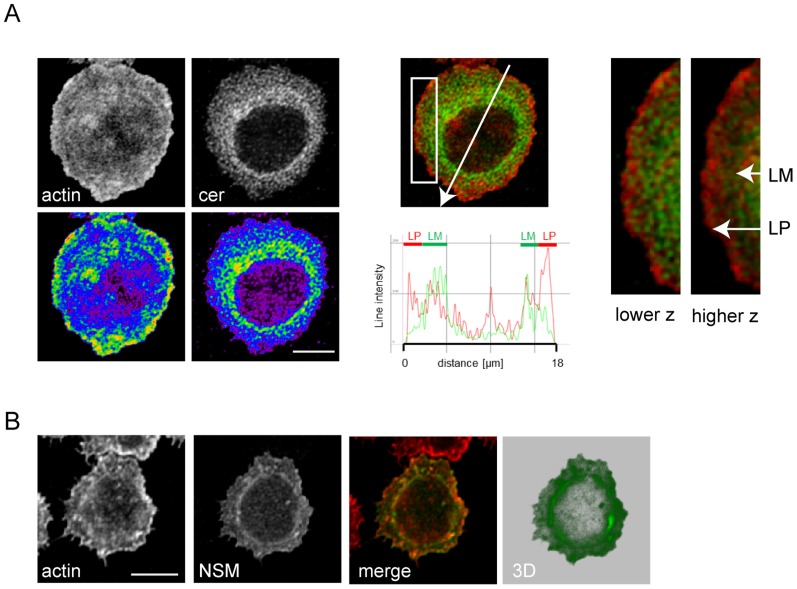
Ceramide and NSM2 accumulate within the lamellum in co-stimulated T cells. A. Ceramide and f-actin were co-detected in T cells 15 min after seeding onto co-stimulatory slides (left panels, with intensities indicated by false colour representation, bottom panels). Size bar: 5 µm. Subcellular distribution of actin (in red) and ceramide (in green) within the lamellum (LM) or the lamellipodium (LP) are representatively shown as profiles (middle panel, intensity profile plane indicated by the arrow) or blow ups imaged at lower or higher z planes (distance approximately 600 nm)(right panels, enlargements of boxed area middle panel). B. NSM2 and f-actin were co-detected 24 h following nucleofection of p-NSM2-GFP (NSM2: green, f-actin: red). Subcellular distribution of NSM2-GFP is shown after deconvolution and 3D reconstruction (right panel). Size bar: 5 µm.

Cross-regulation of ASM and NSM activity upon co-stimulation implies that activation of these enzymes and ceramide release have to be limited, tightly controlled and eventually compartmentalized during T cell activation. To assess the latter question directly, we co-detected ceramide with f-actin in T cells seeded onto co-stimulatory slides after 10 min. After this period, cells had spread on the planar support with typical IS formation where lamellum (corresponding to the pSMAC where centripetal transport of microclusters occurs), lamellipodium (corresponding to the dSMAC with particularly high actin dynamic reorganization) (as detected by f-actin, Fig. 2A) and the actin-free cSMAC (where TCR activity is terminated) can be visualized (Fig. 2A). Most interestingly, ceramide-enriched membrane domains were virtually absent from the lamellipodium, while they were readily detectable within the lamellum (Fig. 2A). Analyses addressing the subcellular distibution of NSM2, the enzyme most likely involved in generating ceramides at the IS (Fig. 1F), could not be performed in fixed cells using antibodies because commercially available NSM-antibodies revealed a broad reactivity in Western blot analyses rendering them unsuitable to obtain reliable staining patterns. We therefore analyzed NSM2 distribution following nucleofection of GFP-tagged neutral sphingomyelinase [27]. As for ceramide, NSM2-GFP appeared to localize mainly to the lamellum (Fig. 2B). However, the enzyme was not entirely absent from the lamellipodium and the cSMAC (Fig. 2B, 3D reconstruction). Also of note, although the protein expressed well in other cell types ([27], [28] and 293 cells, not shown), expression levels of NSM2-GFP in T cells were low Fig. 2B, second panel). Altogether, these data reveal that NSM, but not ASM activation does occur early during co-stimulation, however, ceramide release is spatially confined and excluded from areas of high actin activity.

**Figure 3:**
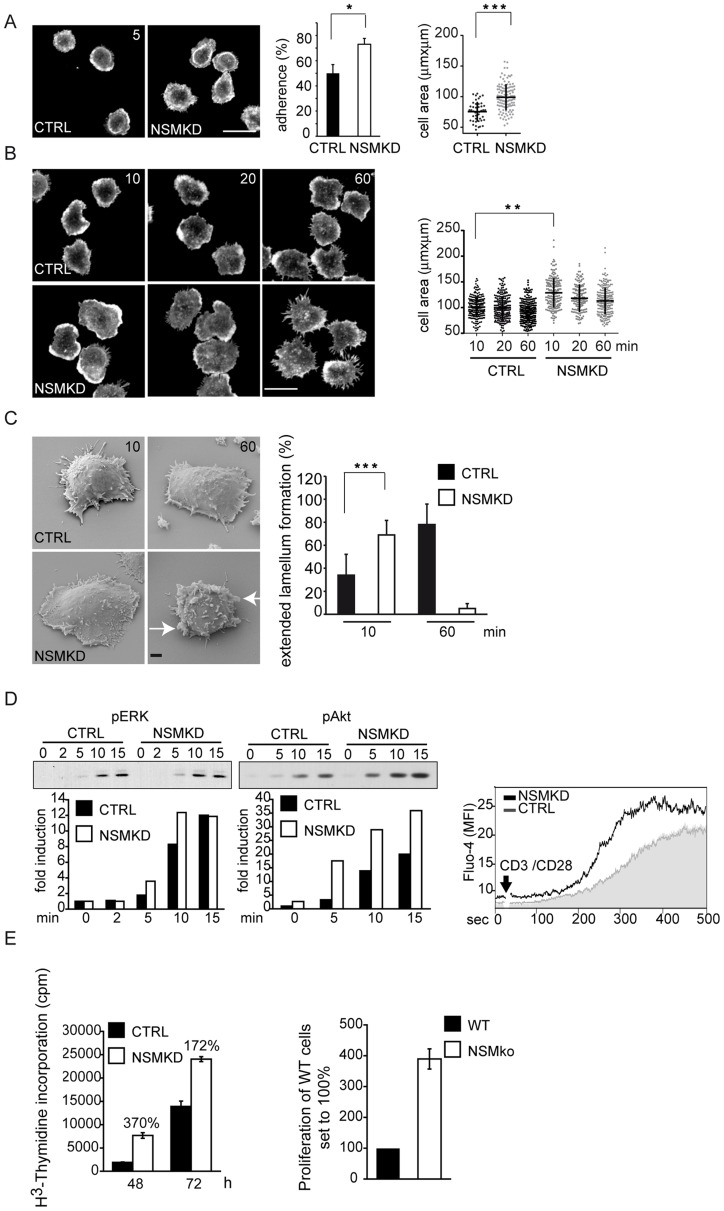
NSM acts to dampen initiation of T cell activation. A–C. T cells transfected with NSM2 (NSMKD) or control siRNA (CTRL) were seeded onto co-stimulatory slides and analyzed for f-actin distribution (A, B), adherence (A), cell area (A, B) and formation of extended lamellar protrusions (C) after 5 min (A) or the time intervals indicated (B,C) by confocal (A, B) or scanning electron microscopy (C; veils marked by arrowheads; at least 50 cells were recruited per culture into the analysis). Representative examples of the quantitative analyses are depicted in the left panels. Size bars: A, B: 10 µm, C, 1 µm. D. Left panels: pERK or pAkt induction following αãCD3/CD28 activation (min) in CRTL and NSMKD T cells (signals for pAkt and pERK and the respective protein loading controls were quantified by AIDA software and are expressed as ‚fold induction', middle panels) Right panel: Ca^2+^ mobilization was determined over time in NSMKD (open histogram) versus CTRL T cells (filled histogram) following α-CD3/CD28 activation by flow cytometry. Representative examples out of at least each 3 are shown. E. Proliferative responses of human primary NSMKD (white bars) or CTRL T cells (black bars) 48 h and 72 h following αãCD3/CD28 activation (left panel) or *Smpd3*-deficient or –sufficient mouse splenocytes 72 h following co-culture with syngenic, superantigen-loaded bone marrow derived DCs (right panel).

### NSM deficiency renders T cells hyper-responsive to stimulation

Early NSM activation during co-stimulation suggested that the enzyme may have a role in T cell activation. To address this, we comparatively analyzed parameters important in early T cell activation in CTRL and NSMKD T cells (which did not detectably differ with regard to surface expression of CD3 or CD28, S1C Fig.). When seeded onto co-stimulatory slides, NSMKD T cells more efficiently adhered and spread than CTRL T cells already after 5 min (Fig. 3A). 10 min following activation (when comparable amounts of CTRL T cells had adhered) NSMKD T cells revealed an enhanced spreading response acquiring extended cell areas framed by actin-dense lamellipodial extensions. Moreover, they detectably polarized (usually representing late stages of activation) giving rise to extended protrusions 20 or 60 min following activation (Fig. 3B). These observations were confirmed by scanning electron microscopy analyses, where NSMKD T cells acquired a flattened, lamellar appearance tightly interacting with the planar support already 10 min following stimulation, which was seen in CTRL T cells only after 60 min. Remarkably, at that time NSMKD, but not CTRL T cells, had developed veil like protrusions indicative for acquisition of a more motile phenotype (Fig. 3C, arrows, and S2A Fig.). This indicates that T cell activation occurs more rapidly upon NSMKD and is associated with enhanced actin cytoskeletal activity. Moreover, accumulation of tyrosine phosphorylated protein species (p-tyr), p-ERK and p-Akt was accelarated and enhanced in NSMKD T cells as was initiation and magnitude of Ca^2+^-fluxing indicating that NSM depletion facilitated early T cell activation (Fig. 3D and, for overall p-tyr, Fig. 6A). Importantly, this also translated into proliferative responses of NSMKD T cells which expanded significantly more efficient in response to CD3/CD28 ligation than CTRL T cells. As seen for spreading responses, NSMKD also appeared to especially support early expansion of co-stimulated T cells (Fig. 3E, left panel, 48 h). As for human cells, proliferation of splenocytes isolated from *Smpd3* deficient *fro/fro* mice driven by syngenic, superantigen-loaded bone marrow derived DCs was significantly enhanced as compared to that of *Smpd3* sufficient littermates (Fig. 3E, right panel). In contrast to expansion, neither release of cytokines (IL-2, IL-4, IL-5, IL-10, IFN-γ or TNF-α) 4, 10, 24 or 72 h following α-CD3/CD28 stimulation or intracellular accumulation of IL-2, IL-10, IFN-γ or IL-17α) following a 4 h restimulation were detectably affected by NSM knockdown in human T cells (not shown). Altogether, these observations suggest that NSMKD facilitates initiation of T cell activation and therefore, NSM activity acts to dampen early T cell activation thresholds.

### MV exposure alters ceramide and NSM compartmentalization at stimulatory interfaces

**Figure 4:**
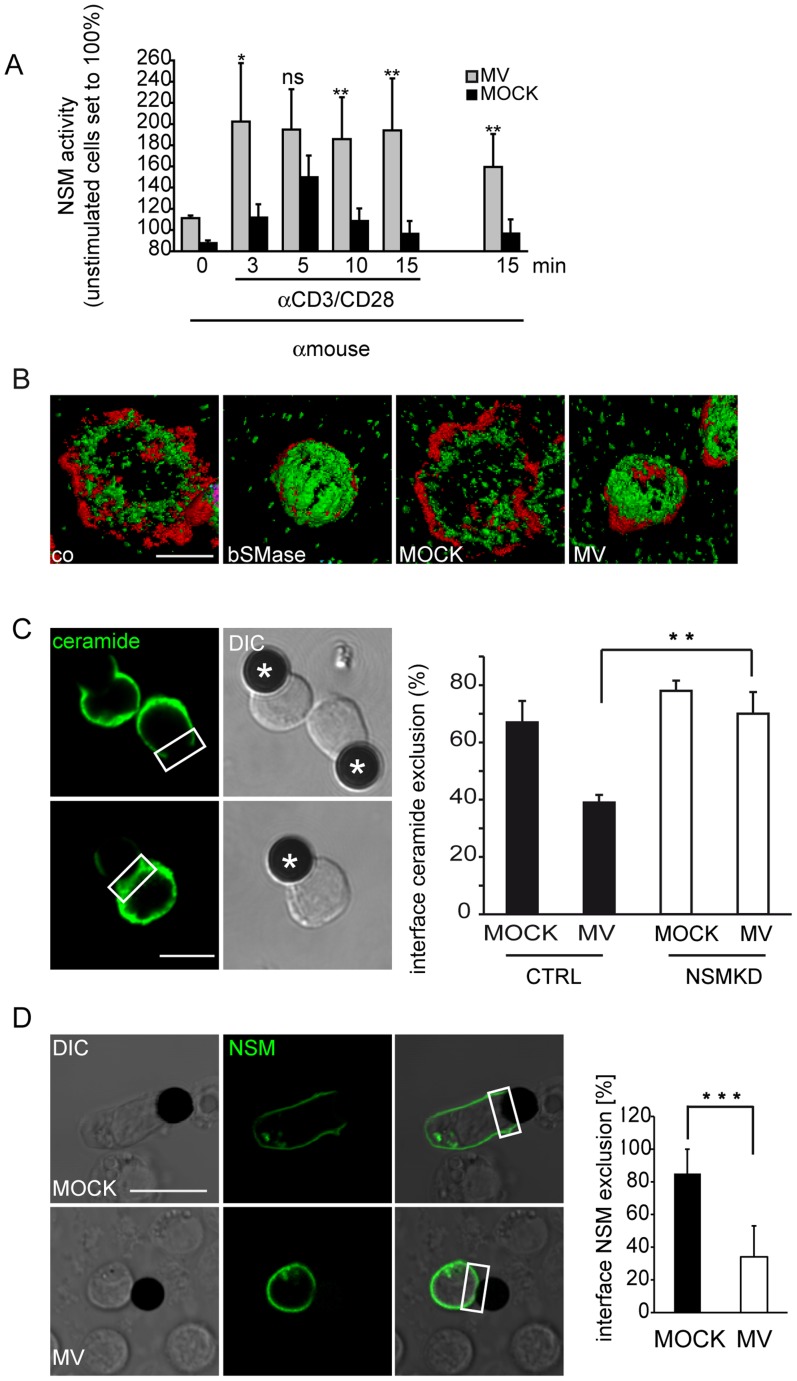
MV causes early superactivation of NSM and ceramide accumulation within stimulatory interfaces. A. NSM activity was determined in membrane extracts isolated from T cells exposed to MV or MOCK prior to αãCD3/CD28 activation for the time intervals indicated. Time point ‚0 and 15 min' (right) measured NSM activity in T cells exposed to MV but not stimulated by αãCD3/CD28 ligation. B. Ceramide (green) and f-actin (red) were detected in T cells pre-exposed to bSMase, MOCK or MV and seeded onto co-stimulatory slides for 15 min. Representative examples of 50 cells analyzed per culture are shown after 3D deconvolution. C. The percentage of conjugates excluding ceramide from the interface between αãCD3/CD28 coated beads (marked by asterisks) and CTRL or NSMKD T cells pre-exposed to MV or MOCK was determined 15 min following conjugate formation, fixation and staining by α-ceramide antibody. D. 24 h following p-NSM2-GFP nucleofection, T cells were exposed to MOCK or MV, conjugated to αãCD3/CD28 coated beads and the frequency of conjugates excluding NSM2-GFP from the interface in both cultures was determined. C, D: Left panels show examples for interface exclusion/inclusion (boxed areas), at least 100 conjugates/per culture were recruited for quantitation in three independent experiments. DIC: differential interference contrast. Size bars: A, B: 5 µm, C: 10 µm.

If NSM activity regulates the initiation threshold of physiological T cell activation, conditions additionally enhancing NSM activity could possibly further dampen T cell activation by promoting timely or spatially aberrant ceramide release. MV is known as an efficient inhibitor of T cell activation and its ability to cause sequential NSM/ASM activation in these cells has been established by us earlier [18]. In line with our previous findings, MV caused NSM activation in T cells (Fig. 4A). When compared to NSM activity induced upon co-stimulation alone, that induced upon additional MV exposure was elevated and persisted indicating that MV supports exaggerated and sustained NSM activation during T cell activation. Because studies involving bacterial sphingomyelinase (bSMAse) or short to middle chain ceramides suggested an inhibitory activity of ceramides in T cell stimulation [23], [24], we assessed whether augmented NSM activation would affect subcellular redistribution of ceramides. When exposed to bSMase or MV (both of which caused comparable ceramide release within 20 min, S3A Fig.), T cells barely adhered and spread on co-stimulatory slides (S2B Fig.). Because of the substantial differences in cell areas, it was not possible to evaluate whether the apparent central concentration of ceramide clusters in bSMase or MV pre-exposed cells resulted from condensation of the contact plane or mis-localization of ceramides (Fig. 4B). As an alternative approach, we analyzed ceramide accumulation at interfaces formed between T cells pre-exposed to MV and co-stimulatory beads coated with CD3/CD28-specific antibodies. In line with our observations made in the planar system (Fig. 2A and 4B), ceramides were largely excluded from the center of the interfaces formed with MOCK treated T cells, and this was unaffected by NSMKD (Fig. 4C). As revealed for adhesion to other supports, MV pre-exposure impaired adhesion of CTRL T cells and thereby the frequency of conjugates with beads (not shown). A substantial fraction of conjugates, that did form with MV-exposed CTRL T cells, however, did not efficiently exclude ceramide from the interface, which was, however, entirely corrected for in NSMKD T cells (Fig. 4C). Thus, MV-induced NSM activation is followed by mis-compartmentalization of ceramide within the IS. To address whether NSM is also excluded from the IS center in this system, and if so, wether this is altered in MV-exposed T cells, we studied distribution of the enzmye in bead assays involving NSM-GFP expressing T cells. In MOCK treated cells, NSM-GFP was detected in association with the plasma membrane, but also with intracellular, presumably Golgi, compartments as described previously for MCF-7 cells [28] (Fig. 4D). Interestingly, these efficiently polarized to the distal pole of the T cell while NSM-GFP was mainly excluded from the bead interface (Fig. 4D, upper panel). In MV-exposed cultures, T cells remained mainly round and failed to detectably polarize which also referred to the localization of the intracellular NSM-GFP enriched compartments (Fig. 4D, bottom panels and S2 Fig.). As seen for ceramides, NSM2 was not efficiently excluded from the interfaces involving MV-pre-exposed T cells indicating that MV signaling interferes with compartmentalization of both NSM2 and ceramides in the IS.

#### NSM ablation corrects for MV-induced interference with T cell adhesion and spreading

**Figure 5:**
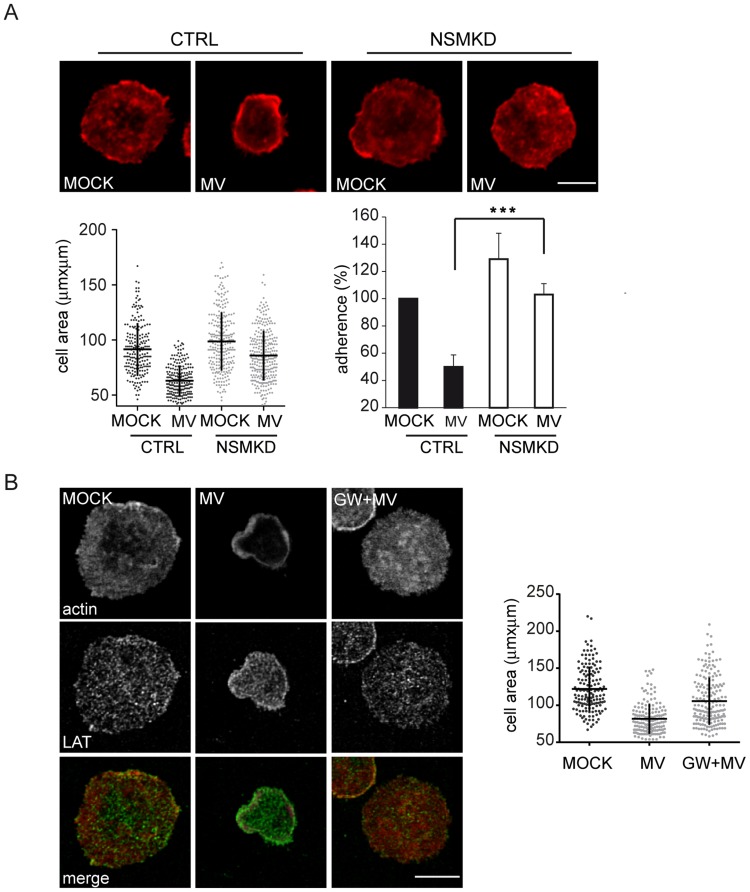
MV-mediated NSM activation accounts for the loss of spreading responses. Cell areas were determined in T cells where NSM activity was genetically (NSMKD, A) or pharmacologically (pre-exposure to GW4869, B) ablated prior to MOCK or MV exposure and subsequent seeding onto co-stimulatory slides for 15 min. Spreading responses were detected by staining for f-actin alone (A) or co-detection with LAT used to detect microclusters (B). Frequencies of CTRL or NSMKD cells adhering to the co-stimulatory slides after exposure to MV or MOCK were determined (A). Size bars: A, B: 5 µm.

In order to identify potential targets of MV-induced NSM activation in T cell suppression, we comparatively analyzed actin dependent spreading responses of CTRL and NSMKD T cells. As seen with co-stimulatory beads, MV pre-exposure interfered with adhesion of CTRL, but not NSMKD T cells, which expectedly, adhered more efficiently (Fig. 5A, compare also Fig. 3A). When cell areas of CTRL or NSMKD T cells pre-exposed to MV or MOCK were comparatively analyzed, genetic NSM ablation slightly, yet detectably resulted in increased cell areas as also noted earlier (Fig. 3, 5A, bottom graph, and S2B Fig.). MV caused a prominent reduction in cell area in CTRL T cells, however, not in NSMKD cells indicating that MV-induced loss of spreading responses was dependent on NSM activation by the virus (Fig. 5A). This interpretation was confirmed upon pharmacological inhibition of NSM by GW4869 where MV treatment substantially reduced the mean cell area in untreated, but not inhibitor pre-exposed T cells as also reflected by redistribution of f-actin and LAT clusters (Fig. 5B, upper panels and graph). Short term exposure to dexamethasone activates NSM2 in other cell types [29], and this is also true for primary T cells where spreading responses are affected upon dexamethasone exposure (S3B Fig.) further supporting the role of this enzyme in this activation parameter also induced by the virus.

### NSM activation contributes to MV interference with T cell activation and expansion

**Figure 6:**
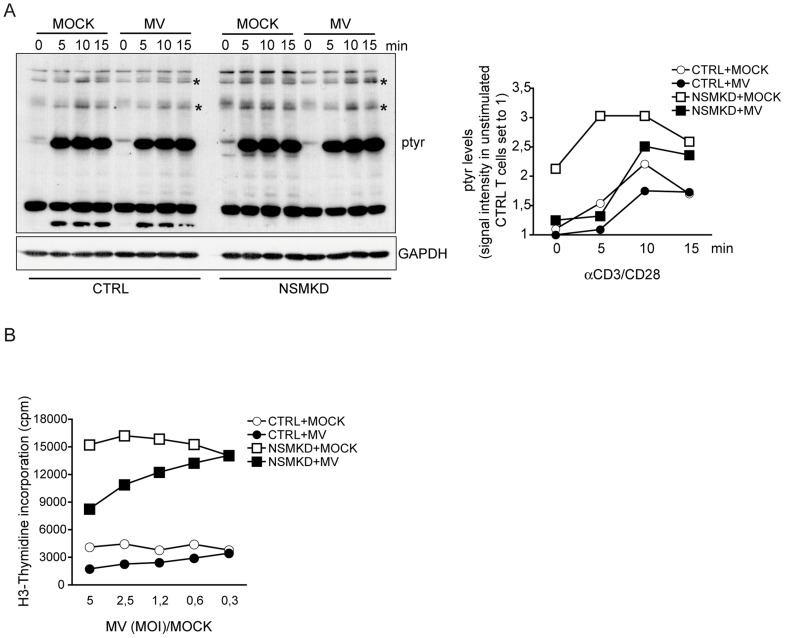
NSM activation contributes to MV interference with T cell early activation and expansion. A. Overall tyrosine phosphorylation was determined at the time intervals indicated following αãCD3/CD28 activation in CTRL and NSMKD cells pre-exposed to MOCK or MV (MOI 1,2) by Western Blotting (where GAPDH detection served as loading control). p-tyr protein species detectably affected by MV interference are starred (*). Right: densitometric analysis of p-tyr profiles of all p-tyr species was performed for each individual lane, quantified using AIDA software and standardized to the signal intensities in unstimulated MOCK treated cells. B. Proliferation efficiencies of CTRL and NSMKD cells pre-exposed to MOCK (each: white symbols) or MV (each: black symbols) at the MOIs indicated (or corresponding protein amounts of MOCK) were determined after 48 h by incorporation of [^3^H]-thymidine.

To evaluate whether defective spreading responses were associated with impairments of T cell activation, accumulation of tyrosine phosphorylated protein species (p-tyr) indicating relay of TCR signaling in response to α-CD3/CD28 stimulation were analyzed in NSMKD T cells pre-exposed to MV or MOCK. In CTRL T cell cultures, MV exposure appeared to compromize accumulation of certain, yet not all p-tyr protein species as compared to MOCK-treated cells (Fig. 6A, left, asterisks). NSM knockdown substantially increased accumulation of p-tyr protein species in MV exposed cells (NSMKD+MV), which, however, still remained below the levels seen in MOCK-treated NSMKD T cells which by far exceeded those seen in CTRL T cell cultures (Fig. 6A, right panel). These data support the overall importance of NSM activity in downmodulating initiation of T cell activation (Fig. 3), but also reveal that MV mediated NSM activation contributes to alterations seen with regard to p-tyr accumulation after TCR triggering. To study the impact of NSM activation on MV-induced inhibition of stimulated T cell expansion, NSMKD and CTRL T cells were exposed to MV (or the corresponding amounts of MOCK) and proliferation was analyzed 48 h following αãCD3/CD28 stimulation. MV dose dependently inhibited T cell expansion both in CTRL and NSMKD T cells, however, the latter expanded substantially more effective indicating that NSM ablation enables high proliferative activity in spite of MV inhibitory signaling (Fig. 6B). Therefore, NSM activation by MV contributes, however, not fully accounts for MV T cell inhibition. Altogether, these findings suggest that NSM activation during TCR triggering acts to dampen T cell activation, which, when inappropriately elicited, results in T cell suppression.

## Discussion

**Figure 7:**
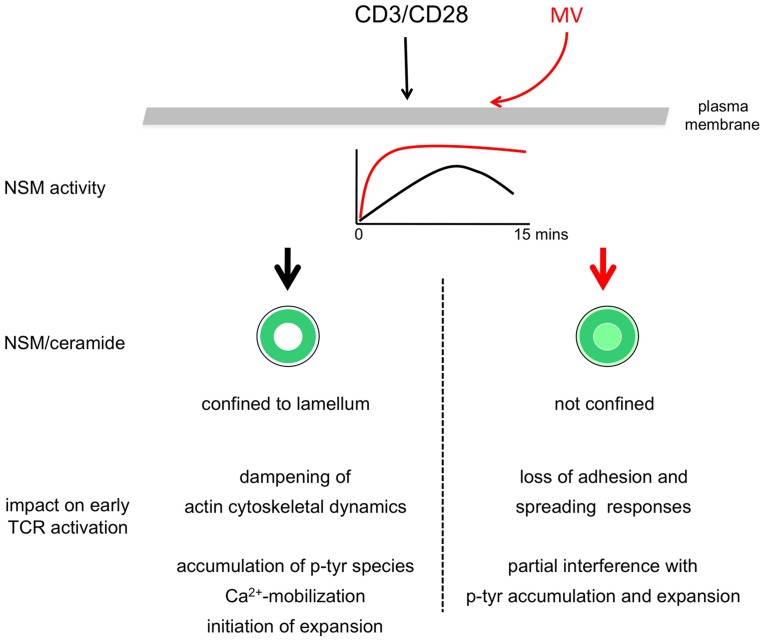
Model of NSM-dependent T cell suppression. T cell co-stimulation induces transient activation of the NSM which, as ceramides, is confined at early stages to the lamellum (upper graph, black line, left sketch of the T cell IS). In the presence of MV, NSM is activated more rapidly and prolonged, and ceramides are not excluded from the IS center (upper graph, red line, right sketch of the T cell IS). Based on genetic ablation studies, under physiologic conditions, the NSM appears to dampen overshooting T cell activation by regulating the speed of early T cell activation as defined by the typical parameters listed. When inappropriately (time, magnitude) activated as occurring by MV T cell contact, the dampening role of NSM is exaggerated and associated with actin cytoskeletal paralysis, but also contributes to MV-induced loss of certain p-tyr protein species and expansion. Therefore, the ability of MV to cause NSM induction in a contact dependent manner accounts for major features of MV-mediated T cell suppression.

Using siRNA mediated genetic NSM ablation, our present study reveals the hitherto unknown activity of the enzyme to dampen the activation of co-stimulated T cells. This is because T cell adhesion, spreading, actin cytoskeletal dynamics, accumulation of tyrosine phosphorylated protein species and expansion are significantly enhanced upon NSM knockdown (Fig. 3, 5–7). At the subcellular level, accumulation of ceramide enriched membrane domains is compartmentalized within the lamellum, whereas ceramides appear to be excluded from the IS center and the lamellipodium (Fig. 2). Indicating that MV exploits and augments the dampening activity of NSM to interfere with T cell activation, MV exposure causes substantial accumulation of ceramide within the IS. This and MV-induced loss of adhesion and spreading responses, is entirely, while MV-interference with p-tyr and T cell expansion are partially rescued upon NSM ablation (Fig. 7). Unfortunately, it is impossible to evaluate the role of the enzyme in MV immunosuppression *in vivo*. Firstly, mice are not permissive for peripheral MV infection and therefore cannot be used to study MV T cell paralysis, and secondly, *Smpd3* deficient animals (also referred to as fragilis ossium (fro/fro) mice) suffer from severe chondrodysplasia and dwarfism which, together with their low birth frequencies (90% of the embryos are lost prenatally) precludes infection experiments [30]–[32].

Ceramide enriched microdomains usually generated in response to stimulated activation of sphingomyelinases and subsequent sphingomyelin breakdown are sites of lateral protein segregation also including receptors and associated signalosomes [3], [33], [34]. Both phenomena efficiently regulate cellular responses to exogenous triggers in a variety of cells also including T lymphocytes where relay of extracellular cues translates into regulation of motility, adhesion and activation all of which are associated with massive reorganizations of the cytoskeleton and signalosome complexes [19], [35]. The role of sphingomyelinases and ceramide release early in TCR activation remained, however, controversal.

While confirming earlier finding that CD3 ligation alone causes NSM activation which peaked late after activation [20], we found that co-stimulation resulted in an early rise of the NSM activity which subsequently returned to background levels. Thus, NSM activity upon CD3/CD28 co-ligation is timely restricted and not prolonged within the first 30 min of stimulation (Fig. 1). As reported earlier [21], ligation of CD28 alone induced ASM and surface ceramide display. This was, however entirely ablated upon co-ligation (Fig. 1), indicating that ASM activity is dispensable for early T cell activation. In line with this, ASM activation or exogenous ceramide supply were not required for or even inhibitory to early TCR signaling and co-stimulation [22], [23]. Indicating that ASM activity interferes with T cell activation, abrogation of TCR-stimulated Ca^2+^ mobilization following CD95 or TNFR-ligation was attributed to ASM activation [18], [36], [37]. In contrast, ASM activation has also been reported to be beneficial for CD28-mediated NF-κB activation or discharge of cytokines from T cells [22], [38]. In co-stimulated cells, early ASM activation did not occur (Fig. 1), and we did not detect any significant impact on NSM ablation on accumulation levels of the cytokines measured intracellularly or in supernatants (not shown). Though NSMKD clearly enhanced kinetics of early T cell activation, activation of CTRL cells to comparative levels occurred (though with delay, Fig. 3,6) and therefore, NSM relating alterations of late activation functions such as cytokine production might not be highly pronounced. Moreover, to the best of our knowledge, except for its role in exosome production [39]–[41], a role of NSM in vesicular trafficking and or discharge of vesicular compartments has so far not been revealed.

NSM/ASM cross-regulation has been reported earlier. Asm was substantially elevated at RNA, protein and activity level in fibroblasts of Smpd3-deficient *fro/fro* mice in the absence of stimulation [26], which was, however, not the case for NSMKD T cells (S1B Fig.). In MV exposed T cells, NSM activity was required for ASM activity triggered by the virus via an unknown receptor [18]. It is beyond the scope of this study to unravel mechanisms underlying NSM/ASM crossregulation in our system, which, however, describes the first example of crossregulation by a receptor complex.

By revealing that NSM ablation clearly had an enhancing effect on steady state actin dynamics, early TCR signaling and expansion, we established the role of the enzyme in regulating kinetics and magnitude of T cell activation (Fig. 3, 6, 7). In contrast to enhanced proliferative responses seen in NSMKD or Smpd3-deficient splenocytes, re-introduction of *Smpd3* corrected for a strongly impaired cell cycling of Smpd3-deficient *fro/fro* fibroblasts [26] indicating that either compartmentalized receptor-mediated activation or cell type specificity defines the impact of NSM on proliferation. In line with our observations made with regard to an inhibitory role of the enzyme in T cells, choleratoxin B mediated inhibition of human CD4 cell proliferation has been related to activation of NSM in lipid rafts [42]. Notably, however, reduced ceramide levels in *fro/fro* fibroblasts were associated with elevated PI3K activity and p-Akt levels indicating a role of NSM in general prevention of activated cellular steady state [43]. In line with this, NSM ablation in T cells elevated basal levels of p-tyr prior to αãCD3/CD28 stimulation (Fig. 6).

Molecular targets of NSM in physiological T cell activation remain unclear. As obvious from the p-tyr analysis, ablation of the enzyme enhanced both kinetics and magnitude of signaling responses which arguably might reflect its role in regulating compartmentalization of signaling microclusters rather than specific downstream targets (Fig. 6). Confinement of ceramides and the the majority of the NSM2 to the lamellum where actomyosin dynamics regulates microcluster transport might indicate a role in communication with cytoskeletal adaptors [44], [45]. In turn, ceramide exclusion from the lamellipodium would be compatible with the requirement of sphingomyelin rather than ceramide at areas of high actin turnover [18], [46], [47]. When overexpressed in MCF-7 cells, a NSM-GFP pool was found to shuttle from the Golgi to the plasma membrane [28]. Similarly, a fraction of NSM appeared to be redistributed from a perinuclear compartment upon T cell stimulation, and, most interestingly, late after stimulation, to be segregated from the IS towards the distal pole altogether indicating that trafficking of this enzyme during T cell activation is tightly controlled (Fig. 4D). Mechanisms underlying NSM trafficking are, however, unknown as yet.

Both ceramides and NSM are excluded from the IS center upon physiological TCR activation, and strikingly, ceramides and NSM2 were shifted to the central IS upon pre-exposure to MV prior to stimulation (Fig. 4C, D). Though this was entirely corrected for by NSM ablation, we cannot rule out that ceramide release there reflected aberrant activation of ASM as found to be activated in MV exposed cells- [18].

Rescue of MV-induced ceramide mis-localization, loss of adhesion and spreading responses by NSM ablation identifies the enzyme as of key importance in pathogen induced suppression of early T cell activation (Fig. 4,5). It certainly also contributes to, but not fully accounts for MV interference with p-tyr accumulation and T cell expansion (Fig. 6). Possibly, identification of specific p-tyr targets of MV (which are rescued upon NSM knockdown, examples marked in Fig. 6A) would be informative in delineating proteins activation of which is required in conferring partial resistence of T cells to MV inhibition.

## Materials and Methods

### Ethics statement

Primary human cells were obtained from the Department of Transfusion Medicine, University of Würzburg, and analysed anonymously. All experiments involving human material were conducted according to the principles expressed in the Declaration of Helsinki and ethically approved by the Ethical Committee of the Medical Faculty of the University of Wuerzburg.

### Cells and virus

Primary human PBMCs were isolated from peripheral blood obtained from healthy donors by Ficoll gradient centrifugation. CD3^+^ T cells (purity ≥90%) were enriched from the PBMC fraction using nylon wool columns and maintained in RPMI 1640/10% FCS. The MV wild-type strain WTF was grown on human lymphoblastoid BJAB cells kept in RPMI 1640/10% FCS and titrated on marmoset lymphoblastoid B95a cells. For exposure experiments, MV (or MOCK preparation obtained from uninfected BJAB cells) was purified by sucrose gradient ultracentrifugation. T cells were exposed to MV or a MOCK preparation in the presence of a fusion inhibitory peptide (Z-D-Phe-L-Phe-Gly-OH; 200 mM in DMSO; Bachem) to prevent infection of T cells.

### T cell stimulation and proliferation assay

1×10^5^ T cells when indicated pre-exposed to GW4869 (2 h, 1,3 µM), recombinant bacterial sphingomyelinase (30 mins, 12,5 mU/ml)(both: Sigma-Aldrich), MV or MOCK (each: 2 h on ice) were pre-incubated with CD3- (clone UCHT-1) and/or CD28-specific antibodies (clone CD28.2) (each 1 µg/ml)(both Beckton-Dickinson Biosciences Pharmingen) on ice, subsequently transferred onto 8-chamber slides for immunostaining (LabTekII, Nunc) or 96 well plates (for proliferation assays) precoated with 25 µg/ml α-mouse IgG (Dianova) (1 h at 37°C) and stimulated for the time intervals indicated at 37°C. For pseudo-IS formation, 2×10^5^ T cells were stimulated for 30 min at 37°C in 100 µl RPMI 1640/0,5% BSA with α-CD3/CD28-coated beads (Dynabeads Human T-Activator CD3/CD28; Invitrogen) at a ratio of 4∶1, captured onto a poly-L-lysine-coated slides (LabTekII, Nunc) and fixed at RT for 15 min in 4% PFA/PBS. For proliferation assays, NSMKD or CTRL T cells exposed to MV or MOCK were stimulated for 48 or 72 h including last 24 h labeling period ([^3^H]-thymidine (Amersham)) and analyzed using a microplate scintillation counter. For mixed leukocyte reaction with murine cells, *Smpd3*-deficient splenocytes were isolated from *Smpd3* knockout *fro/fro* mice (or *Smpd3*-sufficient littermates) and co-cultured with syngenic, superantigen-loaded bone marrow derived dendritic cells (ratio 10∶1) for 5 days.

### Nucleofection and sphingomyelinase activity assay

Nucleofection of human T cells was performed according to the manufacturer's protocol (Amaxa) using pNSM2-GFP [48] (kindly provided by Y. Hannun). 24 h following nucleofection, transfection efficiencies were determined by flow cytometry (the percentage of GFP+ cells ranged between 30 and 45%). For silencing of NSM2, human T cells were nucleofected twice with a two days interval with 400 pmol siRNA targeting human *SMPD3* (NSM2) [49] or, for control, a non-targeting siRNA (Sigma-Aldrich). Cell aliquots were harvested at day 5 for activity assays. On average, knockdown efficiencies were higher than 80% at enzyme activity level (as exemplified in Fig. 1C). When indicated, cells were exposed to dexamethasone (10^−5^ M) (Sigma-Aldrich) or ETOH (used as solvent) for 1 h. ASM or NSM activities were determined as previously described [20] with modifications. 3×10^6^ T cells were disrupted by freeze/thawing (methanol/dry ice) in ASM or NSM lysis buffer (pH 5.2 for ASM and pH 7.4 for NSM). Nuclei were removed by centrifugation for 5 min at 1600 rpm. Post-nuclear homogenate was centrifuged for 1 h at 26 000 rpm in PBS with protease inhibitors for detection of NSM activity and in 100 mM Na-acetate, pH 5,2 for ASM. Cell membrane extracts in ASM or NSM specific lysis buffer were incubated with 1,35 mM HMU-PC (6-hexadecanoylamino-4-methylumbelliferyl-phosphorylcholine) (Moscerdam substrates) as an artificial sphingomyelinase substrate at 37°C for 17 h (final volume 30 µl). Fluorescence reading was performed using excitation at 404 nm and emission at 460 nm according to the manufacturers protocol.

### Detection of ceramide, cytokines and Ca^2+^ fluxing by by flow cytometry

For surface detection of ceramides, T cells were fixed with 1% PFA/PBS for 15 min on ice after the indicated time intervals, stained with primary (a-ceramide; clone MID15B4; Alexis Biochemicals; 1 h, 4°C) followed by a secondary (30 min, 4°C) antibody, and analyzed by flow cytometry (FACS Calibur; Becton Dickinson).

Cytokines (IL-2, IL-4, IL-5, IL-10, IFN-γ and TNF-α) were detected in supernatants 4, 10, 24 and 72 h following aãCD3/CD28 stimulation by cytometric bead assay (CBA) according to the manufacturers instructions (human soluble protein flex sets; BD Biosciences) using DIVA and FCAP Array Software. For intracellular cytokine detection, cells were restimulated 72 h following activation with 40 µM PMA/0,5 µM ionomycin for 4 h in the presence of 35 µM Brefeldin A (all: Sigma). Following a mouse Ig bocking step, cells were stained with α-CD4 (Biolegend), fixed and permeabilized prior to staining with antibodies specific for IL-2 (Pharmingen), IL-10 (eBioscience) IFN-γ and IL-17α (both: BioLegend).

For Ca^2+^-mobilization experiments, T cells (1×10^6^) were washed once and loaded with 1 µM Fluo-4 as cell-permanent AM ester (Molecular Probes) in Hanks balanced salt solution (without CaCl_2_, MgSO_4_, and phenol red) containing 5% FCS and 25 mM HEPES (pH 7.5) according to manufacturers' protocol. α-CD3 and α-CD28 antibodies (5 µg/ml) were added in complete Hanks medium and Ca^2+^ flux was determined by flow cytometry.

### Fluorescence microscopy

T cell activation was stopped by adding 4% PFA (in PBS) for 15 min, permeabilized with 0.1% Triton-X100 for 5 min, blocked with 5% BSA and incubated with primary antibodies (α-ceramide, α-LAT; FL-233; Santa Cruz) diluted in 1% BSA overnight at 4°C. Cells were stained with appropriate Alexa488-conjugated secondary antibody (Invitrogen) for 45 min at RT. F-actin was detected with 488 or 555 Fluorochrom-conjugated Phalloidin (Cytoskeleton). Samples were mounted with Fluorochrome G (Southern Biotech). Confocal Laser Scanning Microscopy (CLSM) imaging was performed using a LSM 510 Meta (Zeiss, Germany), equipped with an inverted Axiovert 200 microscope and a 40x or 63x EC Plan-Apo oil objective (numerical aperture 1.3 or 1.4, respectively) and laser lines 488 and 543. Image acquisition was performed with Zeiss LSM software 3.2 SP2. When indicated, 0.15 µm thick z-stacks were acquired and 3–dimensional reconstructed using LSM software or processed by Huygens deconvolution software (SVI, Hilversum, The Netherlands). Quantification of cell area measurement was calculated using the imaging processing program ImageJ (http://rsb.info.nih.gov/ij/). For live analysis, 24 h following nucleofection of pNSM2-GFP T cells were exposed to MV or MOCK, stimulated with α-CD3/CD28-coated beads, subsequently transferred to 6-channel µ-slide VI (ibidi) and immediately imaged by confocal microscopy.

### Scanning Electron Microscopy (SEM)

For SEM, 3×10^5^ T cells were stimulated as described onto co-stimulatory coverslips (12 mm) in a 24-well plate for the time intervals indicated, fixed by addition of pre-warmed 6.25% glutaraldehyde in 50 mM phosphate buffer (pH 7.2) for 10 min at RT and subsequently at 4°C overnight. After a washing step, samples were dehydrated stepwise in acetone, critical point dried and sputtered with platin/paladium before SEM analysis (Jeol JSM 7500 E).

### Western blot analysis

3×10^6^ CTRL or NSMKD T cells were stimulated for the time intervals indicated by cell-bound α-CD3/CD28 antibodies (1 µg/ml each; Becton Dickinson) and plate-bound α-mouse IgG (25 µg/ml; Dianova) in 12 well cell culture plate, lysed in 200 µl Western blot sample buffer after freezing of samples by −80°C followed by boiling for 5 min. p-tyr were detected using Mouse monoclonal antibodies were used to detect tyrosine phosphorylated protein species (p-tyr) (clone 4G10, Millipore), GAPDH, Akt (Santa Cruz), p-Akt and pERK (both: Cell signaling). Quantification of signal intensities was performed using AIDA software (Raytest).

### Statistical analyses

Overall, data shown were acquired in at least three independent experiments involving individual donors. For statistical analyses of data sets, two-tailed Student's *t* test (*p<0,05, **p <0,005 and ***p<0,0005; ns: non significant) was used for throughout the manuscript. Bars show standard deviations.

## Supporting Information

S1 Figure **A. Representative example of extrafacial ceramide detection after 10 mins on T cells left untreated (unstim) or stimulated with α-CD28, α-CD3 or α-CD3/CD28.** MFI (each upper right corner) and percentage of positive cells are indicated. B. Basal ASM activity in T cell transfected with CTRL (black bar) or NSM siRNA (white bar). C. Surface expression levels (% positive cells, mean) of CD3 or CD28 were determined in CTRL T cells (black bars, values set to 100%) and NSMKD T cells (grey bars: CD3, white bars: CD28) by flow cytometry. A. and B: means of three independent experiments are shown.
(TIF)

S2 Figure **A. CTRL and NSMKD T cells seeded onto co-stimulatory slides for 10 or 60 min were analyzed by scanning electron microscopy.** Overview, size bar: 10 µm. B. CTRL or NSMKD T cells pre-exposed to MV or MOCK were seeded onto co-stimulatory slides for 15 min, fixed and stained for f-actin. Overview, size bar: 5 µm.
(TIF)

S3 Figure **A. Ceramides (% positive cells, mean) were detected on the surface of primary T cells left untreated (set to 100%) or exposed to bacterial sphingomyelinase or MV for 20 min by flow cytometry.** B. Primary T cells were exposed to dexamethasone (dex, 10^-5^ M) or the corresponding amount of the solvent (ethanol) for 1 h and NSM activity levels (left panel) and spreading responses on co-stimulatory slides after 15 min were determined (middle (f-actin staining) and right panels (quantification of cell areas). size bar: 10 µm.
(TIF)
