# Supplementary figures and images for: Neutral Sphingomyelinase in Physiological and Measles Virus Induced T Cell Suppression
Source: PLoS Pathog. 2014 Dec 18;10(12):e1004574. doi: 10.1371/journal.ppat.1004574 (PMC4270778; doi:10.1371/journal.ppat.1004574)

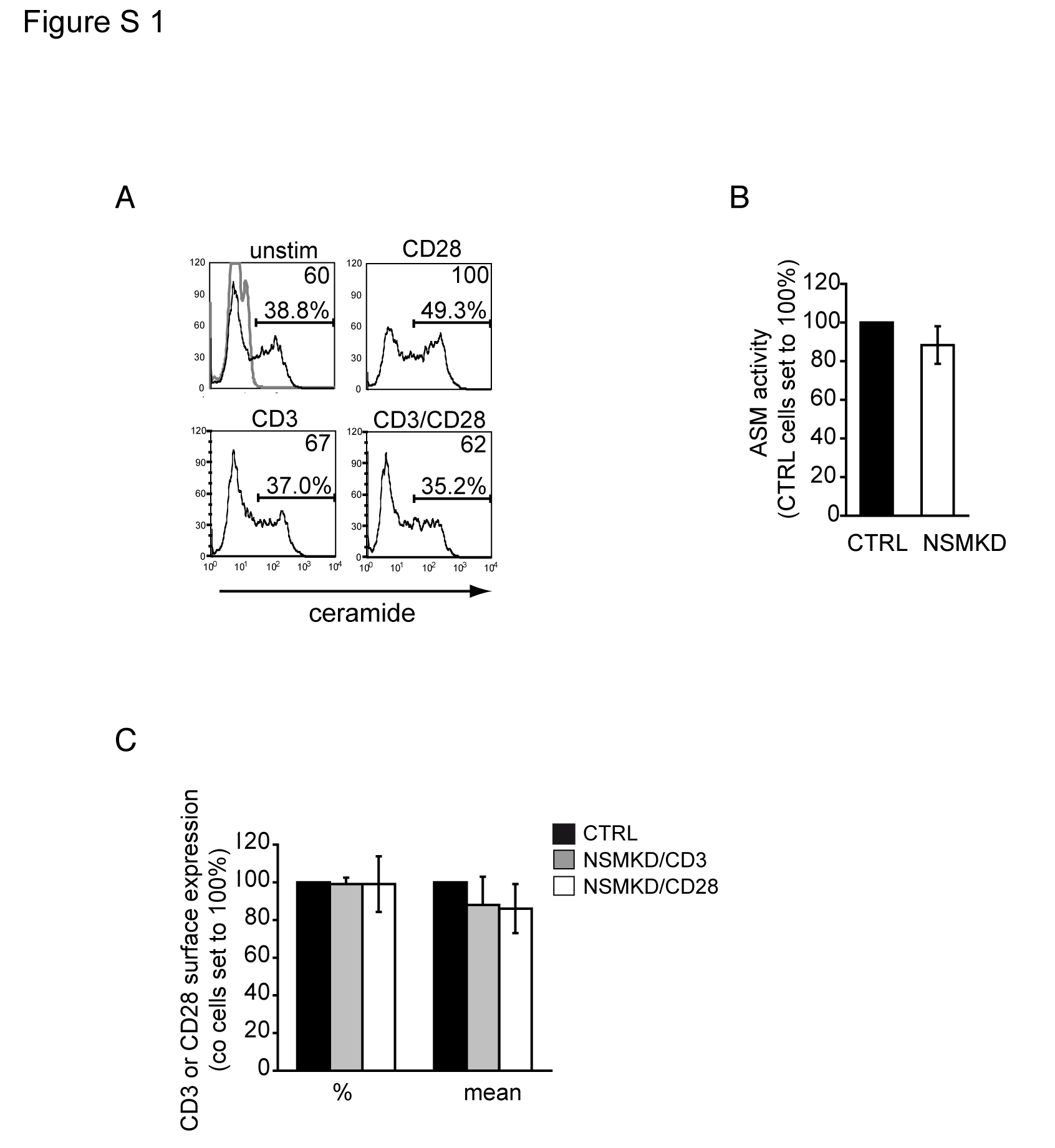

Supplement: S1 Figure — A. Representative example of extrafacial ceramide detection after 10 mins on T cells left untreated (unstim) or stimulated with α-CD28, α-CD3 or α-CD3/CD28. MFI (each upper right corner) and percentage of positive cells are indicated. B. Basal ASM activity in T cell transfected with CTRL (black bar) or NSM siRNA (white bar). C. Surface expression levels (% positive cells, mean) of CD3 or CD28 were determined in CTRL T cells (black bars, values set to 100%) and NSMKD T cells (grey bars: CD3, white bars: CD28) by flow cytometry. A. and B: means of three independent experiments are shown. (TIF) [file ppat.1004574.s001.tif]

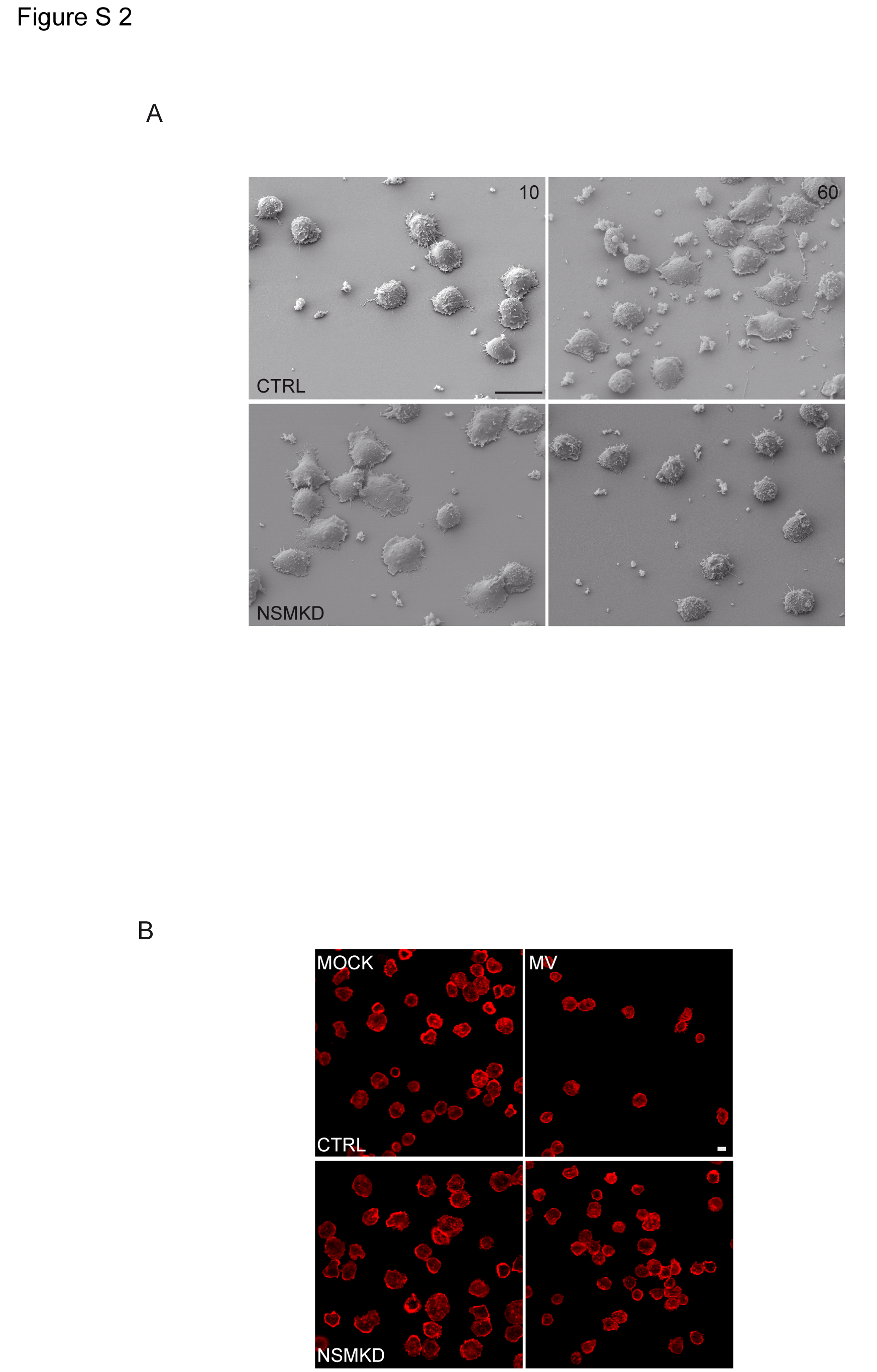

Supplement: S2 Figure — A. CTRL and NSMKD T cells seeded onto co-stimulatory slides for 10 or 60 min were analyzed by scanning electron microscopy. Overview, size bar: 10 µm. B. CTRL or NSMKD T cells pre-exposed to MV or MOCK were seeded onto co-stimulatory slides for 15 min, fixed and stained for f-actin. Overview, size bar: 5 µm. (TIF) [file ppat.1004574.s002.tif]

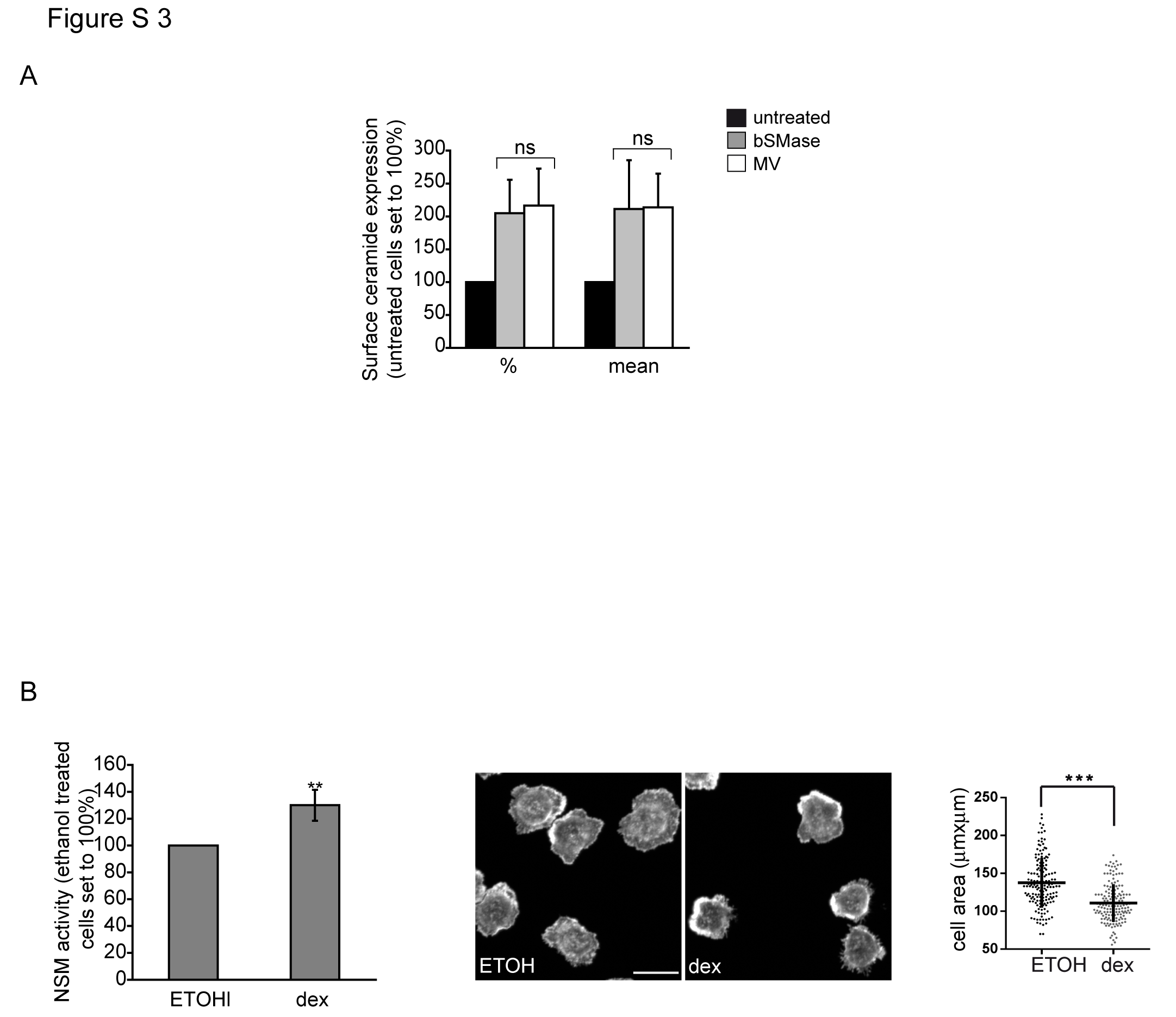

Supplement: S3 Figure — A. Ceramides (% positive cells, mean) were detected on the surface of primary T cells left untreated (set to 100%) or exposed to bacterial sphingomyelinase or MV for 20 min by flow cytometry. B. Primary T cells were exposed to dexamethasone (dex, 10-5 M) or the corresponding amount of the solvent (ethanol) for 1 h and NSM activity levels (left panel) and spreading responses on co-stimulatory slides after 15 min were determined (middle (f-actin staining) and right panels (quantification of cell areas). size bar: 10 µm. (TIF) [file ppat.1004574.s003.tif]
